# Hormonal Modulation of Breast Cancer Gene Expression: Implications for Intrinsic Subtyping in Premenopausal Women

**DOI:** 10.3389/fonc.2016.00241

**Published:** 2016-11-14

**Authors:** Sarah M. Bernhardt, Pallave Dasari, David Walsh, Amanda R. Townsend, Timothy J. Price, Wendy V. Ingman

**Affiliations:** ^1^Discipline of Surgery, School of Medicine, The Queen Elizabeth Hospital, University of Adelaide, Woodville, SA, Australia; ^2^The Robinson Research Institute, University of Adelaide, Adelaide, SA, Australia; ^3^Department of Medical Oncology, The Queen Elizabeth Hospital, Woodville, SA, Australia

**Keywords:** premenopausal breast cancer, intrinsic subtyping, menstrual cycle, gene expression, hormones

## Abstract

Clinics are increasingly adopting gene-expression profiling to diagnose breast cancer subtype, providing an intrinsic, molecular portrait of the tumor. For example, the PAM50-based Prosigna test quantifies expression of 50 key genes to classify breast cancer subtype, and this method of classification has been demonstrated to be superior over traditional immunohistochemical methods that detect proteins, to predict risk of disease recurrence. However, these tests were largely developed and validated using breast cancer samples from postmenopausal women. Thus, the accuracy of such tests has not been explored in the context of the hormonal fluctuations in estrogen and progesterone that occur during the menstrual cycle in premenopausal women. Concordance between traditional methods of subtyping and the new tests in premenopausal women is likely to depend on the stage of the menstrual cycle at which the tissue sample is taken and the relative effect of hormones on expression of genes versus proteins. The lack of knowledge around the effect of fluctuating estrogen and progesterone on gene expression in breast cancer patients raises serious concerns for intrinsic subtyping in premenopausal women, which comprise about 25% of breast cancer diagnoses. Further research on the impact of the menstrual cycle on intrinsic breast cancer profiling is required if premenopausal women are to benefit from the new technology of intrinsic subtyping.

## Introduction

Approximately 25% of breast cancers are diagnosed in women under the age of 50 (1). When breast cancer is diagnosed in young women it carries a high burden, with reduced 5-year survival rates compared to breast cancer in older women (2, 3), and a devastating impact on young families. Breast cancer is considered a chronic disease, with increased mortality extending over the next 40 years, even if the breast cancer is diagnosed at an early stage (3).

In premenopausal women, cyclical production of ovarian hormones estrogen and progesterone occur over the course of the menstrual cycle, causing the mammary gland epithelium to undergo cycles of proliferation, differentiation, and apoptosis (4, 5). Estrogen and progesterone play key roles in the development of breast cancer, with the relative risk of breast cancer related to the breast’s cumulative exposure to these hormones (6, 7).

Breast cancer is not a single disease. There are many mutated genes that can drive tumor development, and biomarkers are essential to classify breast cancer into its different subtypes, each of which responds best to different therapies. Currently, immunohistochemical assays that detect abundance of proteins are used to identify expression of estrogen receptor (ER), progesterone receptor (PR), and human epidermal growth factor receptor 2 (HER2) and determine the rate of proliferation of the cancer cells (Ki67). These biomarkers are used collectively to diagnose subtype and thus determine the best treatment option for an individual patient.

Now, clinics are increasingly adopting gene-expression profiling to diagnose breast cancer subtype, providing an intrinsic, molecular portrait of the tumor. For example, the PAM50-based Prosigna test quantifies expression of 50 key genes to classify breast cancer subtype. Tests that diagnose intrinsic breast cancer subtype must be robust, and relatively resistant to fluctuations in gene expression. Generally, good concordance is observed in gene expression between pairs of diagnostic and surgical samples and between classification of subtype by gene expression and traditional immunohistochemical techniques.

However, despite their availability for diagnosing breast cancer subtype in premenopausal women, tests that utilize gene-expression profiling were largely developed and validated using breast cancer samples from postmenopausal women. Fluctuations in ovarian hormones estrogen and progesterone across the menstrual cycle may affect expression of genes currently used in intrinsic subtyping tests. Indeed, studies have found poor concordance between classification of subtype by intrinsic tests and traditional immunohistochemistry in premenopausal breast cancer patients (8, 9).

Here, we outline the role of ovarian hormones in regulation of gene expression in the breast and highlight the deficiencies in knowledge around intrinsic subtyping in premenopausal breast cancer. As different therapeutic strategies are required depending on the tumor type, effective subtyping of breast cancers is necessary to help guide treatment decisions and provide accurate prognostic information for each patient. Intrinsic subtyping offers some significant advantages over traditional subtyping methods; however, the current tests have not been sufficiently validated in the context of premenopausal breast cancers, where there are significant fluctuations in estrogen and progesterone. There is a pressing need for more research into hormonal modulation of breast cancer gene expression in order to provide the optimal subtype diagnosis for premenopausal women.

## Hormonally Driven Changes in the Breast During the Menstrual Cycle

**Figure 1:**
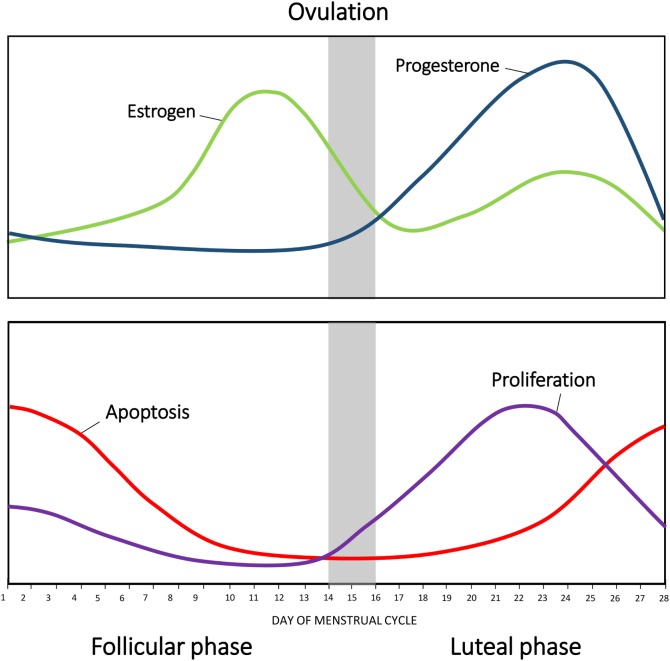
**Changes in hormonal levels in accordance with the menstrual cycle**. The fluctuations of estrogen (green) and progesterone (blue) during the human menstrual cycle. Net apoptosis (red) and proliferation (purple) in the mammary gland in accordance with the menstrual phase.

During the follicular phase of the menstrual cycle, increasing levels of FSH produced by the pituitary stimulate maturation of estrogen-secreting ovarian follicles. Estrogen acts on the pituitary to further increase the production of FSH and LH. Eventually, the concentration of estrogen peaks, stimulating a peak in LH secretion that triggers ovulation. Following ovulation, LH promotes differentiation of the ovarian follicle into the progesterone producing corpus luteum. The luteal phase is characterized by a high concentration of progesterone and is accompanied by a smaller second rise of estrogen. Progesterone supresses FSH and LH production, resulting in a decrease in estrogen concentration. Levels of progesterone begin to decrease as the corpus luteum ceases to produce progesterone and collapses. Consequently, the end of the menstrual cycle is characterized by low circulating hormones, which, in turn, relieve the negative inhibition of FSH and LH (10, 11) (Figure 1), allowing progression into the next menstrual cycle.

Fluctuations in estrogen and progesterone across the menstrual cycle direct the mammary gland epithelium to undergo sequential waves of proliferation, differentiation, and apoptosis (4, 5, 12, 13). Histological analysis of breast tissue by Vogel et al. identified distinct morphological changes in the mammary gland in accordance with different phases of the cycle (14). Five separate phases of breast morphology have been identified, associated with differing concentration of circulating estrogen and progesterone. Each phase has distinct morphological criteria based on the appearances of the luminal cells, myoepithelial vacuolization, intraluminal secretion, stromal edema, and events of cell turnover (5, 14), as summarized in Table 1.

**Table 1:** **Morphological changes in the mammary gland in accordance with the menstrual cycle as described by Vogel et al. (14)**.

Phase 1	Phase 2	Phase 3	Phase 4	Phase 5
		
Follicular phase (days 3–14)		Luteal phase (days 15–27)		Menstrual phase (days 28–2)
*Proliferation*	*Follicular phase of differentiation*	*Luteal phase of differentiation*	*Secretory phase*	*Menstrual phase*
Dense cellular stroma	Dense collagenous stroma	Loose broken stroma	Loose fluid-filled stroma	Dense cellular stroma
Tight closed lumen (no stratification)	Defined lumen (radial orientation)	Open lumen (radial orientation)	Open lumen (radial orientation)	Swollen lumen (radial orientation)
No active secretion	No active secretion	No active secretion	Active apocrine secretion from lumen cell	Rare secretion
High levels of apoptotic bodies	Apoptotic bodies rare	Apoptotic bodies rare	Apoptotic bodies rare	Apoptotic bodies rare

The highest proliferative activity of mammary epithelium is observed in the luteal phase, with rising levels of progesterone (Figure 1). As the concentration of progesterone rises, there is an increase in secondary branching, alveoli budding, and stromal development, accompanied by changes to the extracellular matrix (10, 14). Consistent with this, studies in rodents have shown that administration of exogenous progesterone promotes side-branching and normal secretory alveolar development, whereas estrogen stimulates ductal elongation (15). Ferguson and Anderson showed that epithelial apoptosis increases at the end of the cycle, with decreasing circulating concentration of estrogen and progesterone (16); causing an atrophy of the epithelium, closing of the alveolar lumen, condensation of intralobular stroma, and a variable inflammatory infiltrate (14).

Hormonally driven morphological changes are associated with gene expression changes. Estrogen regulates many genes involved in cell cycle progression; such as Cyclin D (17), and c-MYC (18, 19), and is involved in activation of Cyclin E complexes (20). Estrogen also induces cyclin dependant kinases (Cdk) activation and Rb phosphorylation (20, 21) to promote cell cycle progression. In addition, estrogen treatment inhibits genes responsible for the suppression of cell growth, such as p21 (22). Estrogen is also an inhibitor of apoptosis and increases the expression of antiapoptotic proteins, such as Bcl-2 and Bcl-xL (23). Consistent with this, Bcl-2 is expressed almost exclusively in ER-positive breast cancers and is associated with a good prognosis (24).

Progesterone also plays an important role in cell proliferation and differentiation in the breast, specifically acting during the luteal phase of the menstrual cycle. The proliferative role of progesterone is likely mediated by regulation of cell cycle genes, growth factors, and growth factor receptors. Musgrove et al. illustrated that progesterone treatment of PR-positive breast cancer cells results in an increase in cell cycle progression, which is correlated with an induction of cell cycle genes; including cyclin D1 (25, 26), and c-Myc (25–27). Progesterone also regulates activity of Cdks (28). In addition to stimulating genes associated with cell cycle progression, progesterone has been suggested to inhibit expression of genes responsible for suppression of cell growth, such as tumor suppressor protein p53 (29) and retinoblastoma protein (30). Decline in ovarian hormones also effects gene expression. The fall in estrogen and progesterone at the end of the luteal phase is associated with an increase in apoptotic proteins, such as BAX (10) and FasL (31), and a decrease in antiapoptotic proteins, such as Bcl-2 (10).

In the mammary gland, progesterone elicits its function mainly through a paracrine mechanism. Recently, RANKL has been identified as an important paracrine mediator of progesterone-induced proliferation in the mammary gland (32, 33) and is implicated upstream of Cyclin D (32). Consistent with this, RANKL is required for mammary gland development (34) and was shown to be essential for ductal side branching and alveologenesis in mice (35). In addition, overexpression of its receptor, RANK, in mice resulted in increased proliferation of mammary epithelial cells (36). Wnt-4 has also been identified as a paracrine mediator of progesterone signaling (32, 37), and is important for side-branching of the mammary ductal epithelium (37). To promote optimal proliferation of mammary epithelial cells, estrogen induces expression of the PR. This leads to proliferation of mammary epithelial cells through elevated expression of cell cycle genes, when both estrogen and progesterone are present (38). Conversely, progesterone downregulates its receptor and inhibits synthesis of the ER (39).

**Figure 2:**
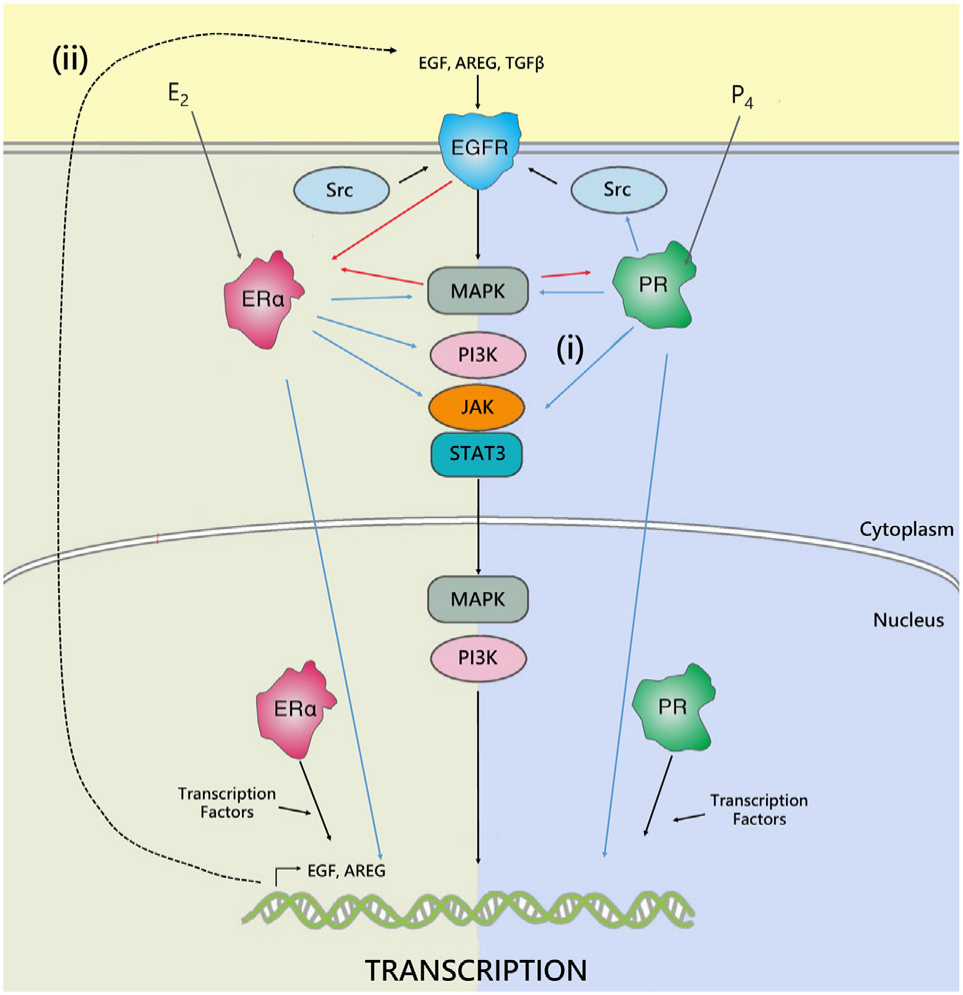
**The interplay between ER, PR, and EGFR**. Hormone receptors regulate gene transcription either by binding directly to DNA response elements or by recruiting transcription factors and co-regulators. In addition, cross talk occurs between ER, PR, and EGFR to regulate gene expression. The estrogen and progesterone receptor can regulate epidermal growth factor receptor activity by either: (i) directly interfering with their transduction pathways, to activate MAPK, JAK/STAT, SRC, PI3K signaling downstream of EGFR, or (ii) by inducing expression and secretion of paracrine growth factors, such as AREG, TGFβ, or EGF, which act on EGFR to activate pathways involved in cell proliferation, survival, and metastasis. In parallel, EGFR can, in turn, phosphorylate and activate ER and PR. Adapted from Tanos et al. (45).

Progesterone and estrogen also regulate growth factors and growth factor receptors in the breast, such as epidermal growth factor (EGF) (40) and EGF receptor (EGFR) (41, 42). Many key genes associated with EGFR signaling are upregulated in response to progesterone treatment (41, 43). Furthermore, EGFR signaling has been implicated downstream of estrogen in the mammary gland (44). Estrogen can induce phosphorylation of EGFR, and can directly interact with signal transduction pathways, to activate MAPK, JAK/STAT, SRC, and PI3K signaling pathways downstream of EGFR. In parallel, EGFR can, in turn, phosphorylate and activate ER and PR (45). It has also been shown that EGF family members are induced by estrogen; including EGF (46), TGFα (46, 47), and amphiregulin (Areg) (48, 49), and act as an important mediator of paracrine estrogen-induced proliferation (Figure 2). However, estrogen inhibits EGFR expression in ERα positive cells. In ERα negative cells, secreted amphiregulin activates EGFR signaling to promote cell proliferation (50). Recent studies have also indicated that estrogen treatment induces expression of vascular epithelial growth factor receptor (VEGF), a receptor involved in tumor growth, both *in vitro* (51) and *in vivo* (52).

In breast cancer, increased EGFR signaling is associated with a more aggressive phenotype. Overexpression of growth factor receptors has been associated with increased metastasis and poor survival, together with a lack of response to endocrine therapy (53, 54). As estrogen and progesterone play critical roles in regulation of growth factors, it is possible that the fluctuations of these hormones during the menstrual cycle are sufficient to modulate expression of EGFR and affect downstream signaling. In the luteal phase when progesterone is high and estrogen is present, signaling through growth factor pathways may be increased compared to the follicular phase when progesterone concentration is low. Consistent with this, breast tumors in young women often have significantly higher EGFR expression and worse prognosis (55, 56).

## Classification of Breast Cancer Subtypes

Breast cancer is a heterogeneous disease, due to its diverse molecular and cellular features, with different therapeutic strategies required depending on the tumor type and stage. The decision to treat patients with adjuvant therapy has been guided by clinical and pathological features of the tumor. With no adjuvant therapy, 12–58% of women will experience a reoccurrence within 5 years (57–59). Of women diagnosed with breast cancer, the majority (approximately 75–92%) receive adjuvant therapy (57, 60, 61), suggesting that many women receive a treatment that may not provide benefit, exposing them to unnecessary side effects. Ideally, the decision to use adjuvant therapy should be based on the prediction of the degree of benefit, to minimize the number of patients receiving unnecessary treatment. Traditionally, evaluation of ER, PR, HER2, and Ki67 immunoreactivity, together with clinicopathological variables including tumor size, type, and grade, are used to classify breast tumors and guide clinical decisions. Breast cancer can be classified into five major subtypes, i.e., Luminal A, Luminal B, HER2 enriched, Basal-like, and normal breast-like, which show significant differences in incidence, survival, and clinical outcomes (9, 62–64).

Luminal A tumors are the most common, representing 50–60% of all breast cancers (65). Patients with Luminal A breast cancer have a good prognosis; displaying significantly increased overall and disease-free survival compared to other breast cancer subtypes (9, 63, 64). Treatment of early-stage Luminal A breast cancer is based mainly on hormonal therapies, with the addition of adjuvant chemotherapy dependant on the clinical stage. The immunohistochemical profile of Luminal A tumors is characterized by high expression of ER, PR, and luminal cytokeratins 8 and 18, an absence of HER2 expression, and low rate of proliferation measured through Ki67 (65, 66).

Luminal B tumors account for 15–20% of all breast cancers (65). Patients with Luminal B breast cancer have poorer outcomes from endocrine therapy, however, have a better response to chemotherapy, achieving pathological complete response (pCR) to neoadjuvant chemotherapy in 16% of tumors compared to 6% in Luminal A tumors (67). From the immunohistochemical point of view, Luminal B tumors are characterized by a lower expression of ER and PR, and higher Ki67 index, and display a higher histological grade, compared to Luminal A tumors (66). Like Luminal A tumors, they express luminal cytokeratins 8 and 18 (65, 66).

Human epidermal growth factor receptor 2-enriched tumors represent 15–20% of breast cancer subtypes (65). Patients with HER2-enriched tumors have poor prognosis and overall survival (9, 63, 64). The immunohistochemical profile of HER2-enriched tumors is characterized by variable ER or PR expression and overexpression of HER2 (66). Consequently, treatment of HER2-enriched tumors includes monoclonal antibodies which directly target the HER2 receptor given in conjunction with chemotherapy (68).

Basal-like tumors comprise 15–20% of all breast cancers (66), and are associated with an aggressive clinical behavior and a high rate of metastasis (69). Patients with Basal-like tumors have a poor prognosis, displaying lower overall and disease free survival compared to other subtypes (9, 63, 64). Treatment of basal-like tumors involves systemic chemotherapy. The immunohistochemical profile of basal-like tumors is characterized by a triple-negative phenotype; ER, PR, and HER2 negative.

Normal breast-like tumors account for 5–10% of all breast cancers (65). They lack the expression of ER, PR, and HER2, however, are not considered basal-like tumors as expression of basal cytokeratin 5 and EGFR is absent (66). However, normal breast-like tumors are poorly defined, and it is argued that they are an artifact of having a high percentage of normal cells in the tumor specimen (70, 71). It has been suggested that these tumors could be grouped into the recently discovered claudin-low subtype, which also displays basal-like characteristics, while also sharing biomarkers in common with normal-like breast epithelial cells. Similar to the basal-like subtype, claudin-low tumors have been associated with therapeutic resistance and poor survival outcomes (72), due to their highly migratory nature.

In clinical practice, identifying triple-negative and HER-2-positive breast cancers can be achieved with standard pathological testing, and recommendations for appropriate adjuvant therapy in early-stage disease are well defined. However, for patients with ER-positive and HER-2-negative disease, distinguishing between those with Luminal A disease and those with Luminal B disease is more challenging and has implications for treatment recommendations (73). Identifying those patients with good prognosis Luminal A disease who will have a small absolute benefit from adjuvant chemotherapy can avoid unnecessary chemotherapy and its associated side effects, while identifying those with Luminal B disease and a higher risk of relapse can prevent under treatment in this group (73, 74).

## Gene-Expression Profiling in Breast Cancer

In 2000, Perou et al. (62) proposed a new classification system for breast cancer subtypes, separating them into distinct subgroups based on gene-expression profiles, as opposed to protein expression signatures used in traditional subtyping methods. Intrinsic subtyping by gene-expression profiling is predictive of overall and relapse-free survival (8, 9, 63, 64, 75) and can predict the relative risk of relapse and the patient benefit from hormonal therapy and chemotherapy (71). Therefore, gene-expression profiling can be used to inform risk prediction and help guide treatment decisions, to decrease the number of patients receiving unnecessary treatment. The main genes associated with each subtype, together with pathological characteristics and prognosis, are summarized in Table 2.

**Table 2:** **Summary of clinical and pathological characteristics, prognosis, and gene-expression changes of breast cancer subtypes**.

Subtype		Biomarker profile					Prognosis				Treatment
	Incidence (%)	ER	PR	HER-2	Ki67	Other	OS (%)	5 years DFS (%)	10 years DFS (%)	Gene-expression changes	
Luminal A	50–60	+	+	−	Low	Luminal epithelial cytokeratins 8 and 18	89–95	79–85	70–78	Increased expression in genes associated with ER function: *FOXA1, PgR, BCL2, EsR1, LIV1, ZIP6, SLC39A, XBP1, GATA3, ERBB3/4*, and TFF1	Hormonal therapies +/− chemotherapy
						Low histological grade					

Luminal B	15–20	+	+	−	Mod	Luminal epithelial cytokeratins 8 and 18	71–85	60–75	50–60	Increased expression in genes associated with ER function: *FOXA1, PgR, BCL2, EsR1, GATA3*	Poorer outcomes from hormone therapy (Low levels of HRs); better pCR to neoadjuvant chemotherapy
						High histological grade				Increased expression of proliferative genes *CCNB1, CCND1, CCNE1, MYBL2*, MKI*67, v-MYB*	

HER-2	15–20	−	−	+	High	Luminal cytokeratins	43–78	41–65	45–51	Amplification of *ERBB2* and GRB7	HER-2-targeted therapy and chemotherapy
										PI3K pathway activation (AKT, pS6, and p4EBP1) correlated with *INPP4B* and *PTEN* loss	
										Increased expression of proliferative genes *BIRC5, CCNE1, CCND1, ORC6L, MYBL2, MKi67*	

Basal-like	15–20	−	−	−	High	Basal cytokeratins 5, 14, and 17	53–73	48–72	48–65	Increased expression of EGFR	Chemotherapy
						High EGFR				Dysregulation of MAPK/AKT/PI3K and Ras/Raf/ and JAK/STAT	Future: EGFR (Gefitinib/Cetuximab), VEGF, or AR inhibition
										Increased expression of *FOXM1, cMYC, CCNE1, CCND1, CDC20, CDC6, BIRC5, ORC6L*	

Claudin-low	12–14	−	−	−	Low	Low luminal markers and high mesenchymal markers	×	~67	×	Loss of tight junction proteins: *claudin 3*,*4*,*7, E-cadherin* and *CDH1* (and highest expression of transcript repressors of *CDH1 vimentin, SNAI1 and 2, TWIST1/2, and ZEB1/2*)	Chemotherapy
										Enrichment for EMT markers: *SNAI1/2, TWIST1/2, ZEB2*	

Normal-like	5–10	−	−	−	High	Negative for CK5 and EGFR	~93	79–87	~85	Loss of tight junction proteins: *claudin 3*,*4*,*7, E-cadherin*	Chemotherapy

Reference	(65, 66)	(66)			(66)	(66)	(9, 67, 72, 76, 77)			(65, 66, 71, 72, 78)	(66)

*The italics refers gene names*.

Each subtype displays a distinct gene-expression profile. Luminal A tumors are characterized by a high level of ER, and as such display an increased expression in genes associated with ER function, such as *Bcl2, EsR1, PgR*, and *FOXA1* (62, 63). Compared to Luminal A, Luminal B tumors display an increase in expression of proliferative genes, and consequently possess a more aggressive phenotype, higher proliferative index, and worse prognosis (9, 63, 64, 67, 79). Like Luminal A tumors, Luminal B tumors also express genes associated with ER activation, including *Bcl2, FOXA1, CCND1*, and *GATA3* (79); however, increased expression of proliferative genes, such as *CCNB1, CCND1, CCNE1, MYBL2*, and *MKi67*, appears to be the hallmark of luminal B tumors (63, 64, 79).

HER-2-enriched tumors are characterized by a high expression of human epidermal growth factor receptor-2, *ErbB2*, and other genes associated with the HER2 pathway (65, 80). HER2 functions as a receptor tyrosine kinase and signals through PI3K/AKT/mTOR, JAK/STAT, MAPK and Ras/Raf pathways to promote cell survival, proliferation, and migration (66). Consequently, a dysregulation of signaling in HER-2-enriched tumors can lead to sustained proliferative signaling, a hallmark of cancer. HER-2-enriched tumors also overexpress *GRB7*, an adaptor protein involved in receptor tyrosine kinase signaling, which can also promote activation of PI3K/AKT/mTOR, JAK/STAT, and MAPK signaling pathways to allow for sustained proliferative signaling (66). HER-2-enriched tumors have increased expression of proliferative genes, including *BIRC5, CCND1, CCNE1, ORC6L*, and *MKi67*, and are often associated with a more aggressive and highly proliferative tumor (80).

Basal-like tumors express high levels of basal cytokeratins 5, 14, and 17, and do not express ER, PR, and HER2. Consequently, basal-like tumors cannot be treated with many conventional therapies, however, have a better response to chemotherapy compared to other subtypes. The EGFR is often overexpressed in basal-like tumors, where increased EGFR expression correlates with poor patient survival. Basal-like tumors display a dysregulation in PI3K/AKT, JAK/STAT, and ERK/MAP signaling pathways and a high expression of proliferative genes, such as *FOXM1, c-MYC, CCNE1 BIRC5*, and *CCND1* (66). In addition, basal-like tumors overexpress genes involved in the progression through the cell cycle (*CDC20, CDC6*) (66) and genes associated with the EGFR pathway (43). Absence of ER expression results in low expression of estrogen-related genes, *EsR1, Bcl2*, and *PgR* (66).

Claudin-low tumors are enriched for epithelial-to-mesenchymal transition markers, such as *SNAI1/2, TWIST1/2*, and *ZEB2* (72), with low expression of tight junction proteins, such as claudin 3, 4, and 7, E-cadherin, and *CDH1* (72). The claudin-low subtype is highly migratory and therefore has a poor prognosis (76).

### PAM50

**Figure 3:**
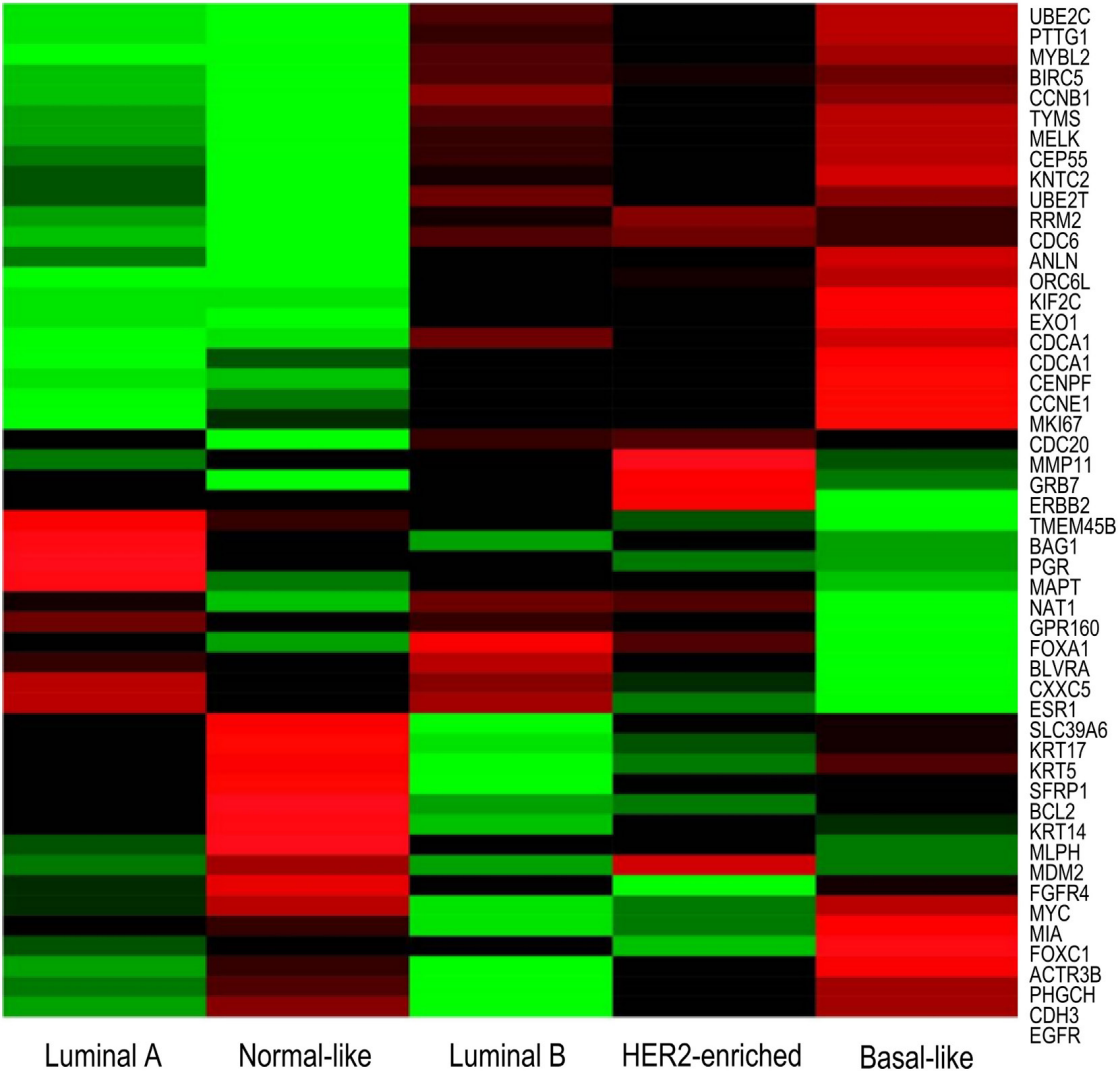
**Microarray heatmap of PAM50 genes expression in “intrinsic” breast cancer subtypes**. Molecular profiles have distinct gene expression. Expression values of genes included in the PAM50 signature are shown as red/green according to their relative expression level for each subtype. Highest gene expression (red), lowest (green), and average (black) (71).

PAM50 is a list of 50 genes that classify breast cancers into one of five intrinsic subtypes from formalin-fixed, paraffin-embedded tissues by real time polymerase chain reaction (RT-PCR) (62). These 50 genes identified were refined from a list of 1,906 genes, which were found in four previous microarray studies. The list was minimized to genes that have passed previously established formalin-fixed paraffin-embedded (FFPE) performance criteria, and were further refined through statistical analyzes, allowing for identification of genes which showed a highest correlation to each intrinsic subtype. Differential gene expression between subtypes is shown by microarray in Figure 3. Subsequent studies have shown that classification of intrinsic subtypes using the PAM50 test retains the prognostic and predictive significance characteristic to breast cancer subtypes (9, 70, 74, 81, 82). Furthermore, several studies have shown that the PAM50 classification method provides better information on prognosis than immunohistochemistry-based surrogates (8, 9, 64). This suggests that subtyping by immunohistochemistry is inferior to genomic profiling, identifying a requirement for gene-expression profiling in a clinical setting.

Accurate testing of predictive biomarkers is important, as discrepancies between IHC and intrinsic classification of breast tumors may lead to differences in treatment decisions and patient outcomes. To address this, ER, PR, and HER2 concordance between immunohistochemistry and gene profiling has been investigated. A number of studies have reported a discordance of below 10% for ER and PR status (8, 9, 70, 83, 84). Cheang et al. evaluated concordance in subtype classification between PAM50 and immunohistochemistry, finding that of Luminal tumors as defined by PAM50, 8% did not stain positive for ER through immunohistochemistry (8). Consistent with this, Chia et al. identified 8% of Luminal tumors instead being classified as either HER-2-positive, or triple-negative through immunohistochemistry (9). As patients with ER negative or HER-2-positive tumors will receive chemotherapy, any discordances in classification of luminal tumors can have critical implications on treatment options, where women may receive unnecessary chemotherapy with no benefit.

Although several studies have reported high overall concordance in HER-2 expression between gene profiling and immunohistochemistry (85–88), others report low concordance (70, 81). Chia et al. (9) found that of HER-2-enriched tumors identified by PAM50, only 66% of these stained positive for HER2 expression by immunohistochemistry. Instead, 31% of HER2-enriched tumors were classified as Luminal A or B tumors, and 4% classified as basal-like through immunohistochemistry. Cheang et al. (8) also identified a low concordance of 69% in HER2 status between PAM50 and immunohistochemistry, with 6% of tumors instead classified as Luminal A tumors, and 16% as triple-negative.

As different subtypes each respond best to different treatments and the absolute benefit of adjuvant therapies depends on the risk of relapse, this poses the question; in discordant cases which result should be used to guide treatment decisions, and is it appropriate to deny a patient treatment that would otherwise be indicated by a different subtyping method? Studies by Chia et al. (9) and Cheang et al. (8) included only premenopausal women and described low concordance between immunohistochemistry and gene profiling. This low concordance may be due to the fluctuations in circulating hormones during the menstrual cycle and the relative effect of hormonal stimulation on gene versus protein expression. While it is believed that PAM50 is more reflective of the true biology of the tumor than protein-based immunohistochemistry, the paucity of data on premenopausal women makes it difficult to determine the efficacy of the PAM50 test compared to the traditional gold standard for tumor subtyping.

Prosigna is an *in vitro* diagnostic assay which is based on the PAM50 gene signature assay. The Prosigna test is performed on FFPE tissue and identifies the patient’s risk of distant reoccurrences of disease; it aims to aid clinicians and patients in treatment decisions. The development of Prosigna from PAM50 is summarized in Table 3. Of note, the validation of the Prosigna test – necessary for FDA approval – was based on two clinical trials (the TransATAC and ABCSG-8 clinical trials) incorporating data from over 2,400 postmenopausal women enrolled in adjuvant aromatase inhibitor trials. Although studies have examined the use of PAM50 in managing adjuvant therapy in premenopausal breast cancer (8, 9), there have been no large-scale clinical trials into the efficacy of the Prosigna test for premenopausal women. Thus, the accuracy of the Prosigna test has never been properly validated in the context of the hormonal fluctuations that occur during the menstrual cycle in premenopausal women.

**Table 3:** **Development of Prosigna, a PAM50-based subtype classifier**.

		Menopausal status			Receptor status	
Reference	Total	Premenopausal	Postmenopausal	Unknown	ER +	ER−
**Development of prosigna**						
Parker et al. (71)	761	–	–	761	544	195
Neilsen et al. (81)	786	20	752	14	768	9
Bastien et al. (70)	154	49	101	4	100	49
Chia et al. (9)	398	398	0	–	291	107
Cheung et al. (8)	476	476	0	–	300	168
Martin et al. (89)	820	443	377	–	645	172
Liu et al. (90)	1094	757	337	–	638	456
Nielsen et al. (91)	43	–	–	15	43	0
Sestak et al. (92)	2137	0	2137	–	213	0
Wallden et al. (82)	746	91	433	222	547	177
**Clinical validation of prosigna**						
TransATAC (93)	1007	0	1007		1007	0
ABCSG-8 (74)	1478	0	1478		1464	17

*Following the development of a 50-gene subtype classifier by Parker et al. in 2009, subsequent studies by the same group clinically and analytically validated the prognostic value of the 50-gene signature. TransATAC and ABCSG-8 trials provided evidence of the clinical validity of Prosigna. Currently in recruitment, is a study evaluating the treatment impact of Prosigna. The numbers of pre- and postmenopausal women included in the studies are indicated. In studies where menopausal status was not given, women under the age of 50 were defined as premenopausal and women over the age of 50 as postmenopausal*.

### Oncotype DX

Oncotype DX evaluates the expression of 21 genes associated with tumor proliferation, invasion, and estrogen signaling (94) (Table 4). In 2004, Paik et al. selected 250 candidate genes from published literature and genomic databases that have been shown to be correlated with disease outcome. The list of genes was reduced to 16 genes, which showed the highest correlation to distant recurrence after 10 years. Relative expression of these genes, in relation to expression of five reference genes, provide a reoccurrence score which is significantly correlated with likelihood of breast cancer reoccurrence in 10 years (94–98). Oncotype DX, therefore, impacts adjuvant treatment decisions and influences treatment recommendations. The development of Oncotype DX is summarized in Table 5. Like the PAM50 gene set, the genes used in the Oncotype DX test rely heavily on genes related to ER and growth factor signaling and proliferation, which are differentially expressed by normal breast epithelial across the menstrual cycle, as discussed above.

**Table 4:** **Panel of 21 genes used in the Oncotype DX assay to determine the risk of distant recurrence**.

Proliferation	Invasion	HER-2	Estrogen	Other	Reference
*Ki67*	*MMP11*	*GRB7*	*ER*	*GSTM1*	*ACTB*
*STK15*	*CTSL2*	*HER-2*	*PgR*	*CD68*	*GADPH*
*Survivin*			*BCL2*	*BAG1*	*RPLPO*
*CCNB1*			*SCUBE2*		*GUS*
*MYBL2*					*TRFC*

*Genes are grouped on the basis of function, correlated expression, or both. The recurrence score is derived from gene expression normalized to reference genes*.

**Table 5:** **The development of Oncotype DX, a 21-gene assay which identifies patient benefit from chemotherapy**.

		Menopausal status		Receptor status	
Reference	Total	Premenopausal	Postmenopausal	ER +	ER−
**Development of oncotype DX**					
Paik et al. (94)	668	194	474	668	0
Esteva et al. (99)	149	122	27	103	46
Gianni et al. (100)	89	–	–	52	31
Habel et al. (101)	790	209	581	682	108
Albain et al. (102)	367	0	367	367	0
**Clinical validation of oncotype DX**					
NSABP B20 (97)	651	289	362	651	0
E2197 (103)	465	193	272	465	0
NSABP B14 (96)	1023	298	725	1023	0
TransATAC (93)	1231	0	1231	1231	0
Tailorx (104)	1623	480	1143	1621	5

*Following the identification of 21-genes which showed high correlation to distant reoccurrence of breast cancer at 10 years, subsequent studies verified its predictive and prognostic value. The numbers of pre- and postmenopausal women included in the studies are indicated. In studies where menopausal status was not given, women under the age of 50 were defined as premenopausal and women over the age of 50 as postmenopausal*.

### EndoPredict

EndoPredict is an RT-PCR-based diagnostic test which evaluates the expression of eight proliferative and hormone receptor-associated genes. In conjunction with the tumors clinicopathological factors, it identifies the risk of distant metastasis within 10 years (105). EndoPredict is used to guide treatment decisions for both chemotherapy and hormonal therapy in ER-positive, HER-2-negative breast cancer.

The EndoPredict gene signature was identified from gene expression profiles of breast cancer samples taken predominantly from postmenopausal women (105). Similar to Prosigna, initial clinical validation of EndoPredict was based on two clinical trials (ABCSG-6 and ABCSG-8 clinical trials), which incorporated data exclusively from postmenopausal women, who were enrolled in aromatase inhibitor trials (105). In 2014, results of EndoPredict prognostic validity from a third clinical study were published. This study included both pre- and postmenopausal women, 54 and 46% of patients, respectively, and suggested that EndoPredict is prognostic in both pre- and postmenopausal women with breast cancer (106). The development of EndoPredict is summarized in Table 6. There are a lack of studies which validate the efficacy of EndoPredict in premenopausal women, and whether EndoPredict is an appropriate tool for guiding treatment decisions in premenopausal women has not yet been sufficiently investigated.

**Table 6:** **The development and clinical validation of EndoPredict**.

		Menopausal status		
Reference	Total	Premenopausal	Postmenopausal	Unknown
**Development of EndoPredict**				
Filipits et al. (105)	964	245	589	
Muller et al. (107)	80	–	–	80
Dubsky et al. (108, 109)	1702	0	1702	
Muller et al. (110)	167	–	–	167
Martin et al. (106)	566	300	255	
Buus et al. (111)	928	0	928	
**Clinical validation of EndoPredict**				
ABCSG-6 (105)	1324	0	1324	
ABCSG-8 (105)	378	0	378	
GEICAM-9906 (112)	566	300	255	

*The numbers of pre- and postmenopausal women included in the studies are indicated. In studies where menopausal status was not given, women under the age of 50 were defined as premenopausal and women over the age of 50 as postmenopausal*.

### MammaPrint

MammaPrint is a diagnostic test which evaluates the expression of 70 genes associated with metastasis, proliferation, invasion, survival, and angiogenesis (113). The list of 70 genes was identified from whole-genome expression arrays, and selected for on the basis of those which were significantly correlated with disease outcome (113). Interestingly, MammaPrint does not measure expression of commonly used diagnostic markers ER, PR, or HER2.

Through the relative expression of these 70 genes, MammaPrint classifies tumors into high or low risk groups, which corresponds with patient’s clinical outcome. Studies have shown that risk groups identified by MammaPrint correspond with patients overall survival (114), disease-free metastasis (75, 115), and the benefit from adjuvant chemotherapy (116, 117). Most studies validating the diagnostic capabilities of MammaPrint were small-scale retrospective studies, which included both pre and postmenopausal women, as summarized in Table 7. MammaPrint had initially been developed and validated in patients under the age of 55, suggesting that MammaPrint is targeted toward the younger population. Furthermore, the first prospective study which evaluated the impact of MammaPrint in assisting with treatment decisions included patients under the age of 55 (118, 119). As MammaPrint was developed in a younger population, and does not measure the gene expression of ER or PR, it is likely that MammaPrint is appropriate for diagnosing breast cancer in premenopausal women.

**Table 7:** **The development of MammaPrint**.

		Menopausal status		
Reference	Total	Premenopausal	Postmenopausal	Unknown
**Development and validation of MammaPrint**				
van’t Veer et al. (113)	97	66	31	
Van de Vijver et al. (75)	295	246	49	
Buyse et al. (115)	302	203	99	
Bueno-de-Mesquita et al. (118)	427	292	135	
Wittner et al. (120)	100	24	76	
Bueno-de-Mesquita et al. (114)	123	83	40	
Mook et al. (121)	241	125	116	
Mook et al. (122)	148	0	148	
Knauer et al. (117)	541	231	310	
Straver et al. (116)	167	119	39	9
Drukker et al. (119)	427	292	135	
Drukker et al. (123)	295	246	49	
Cardoso et al. (124)	6693	2226	4467	

*The numbers of pre- and postmenopausal women included in the studies are indicated. In studies where menopausal status was not given, women under the age of 50 were defined as premenopausal and women over the age of 50 as postmenopausal*.

In 2016, results from a second prospective study were published, comparing MammaPrint to clinicopathological tools for selecting patients for adjuvant chemotherapy (124). The median age of the patients was 55 years. The study found that approximately 46% of patients, who were classified as high risk by clinicopathological features, were also classified as a low risk of metastasis by MammaPrint. Although these tumors presented with a high clinical risk, the results from the study suggested that these patients received no significant benefit from chemotherapy. Therefore, the authors concluded that using MammaPrint to guide treatment can reduce the number of patients receiving unnecessary chemotherapy.

### Breast Cancer Index

The Breast Cancer Index is an RT-PCR based assay which classifies patients into risk groups to predict the likelihood of benefit from endocrine therapy, and the risk of early or late recurrence (125–128). The Breast Cancer Index evaluates two independent biomarkers; the HOXB13:IL17BR gene ratio, which is associated with endocrine therapy response (129), and the molecular grade index, which is determined by the expression of five proliferative-related genes (130). Classification of breast cancer through the expression of these seven genes aims to identify patients which are most likely to benefit from adjuvant therapy. The development of the Breast Cancer Index is summarized in Table 8. Similar to Prosigna, the clinical validation of the Breast Cancer Index was based on retrospective studies which used samples exclusively from postmenopausal women and, therefore, results cannot be generalized to premenopausal women.

**Table 8:** **The development of the Breast Cancer Index**.

		Menopausal status		
Reference	Total	Premenopausal	Postmenopausal	Unknown
**Development and validation of the Breast Cancer Index**				
Ma et al. (131)	80	2	78	
Ma et al. (130)	836	81	327	428
Jankowitz et al. (132)	265	80	185	
Jerevall et al. (128)	588	0	588	
Mathieu et al. (133)	150	66	84	
Sgroi et al. (126)	665	0	665	
Zhang et al. (125)	958	0	958	
Habel et al. (134)	608	162	446	
Sanft et al. (135)	96	13	76	
Sgroi et al. (127)	292	0	292	

*The numbers of pre- and postmenopausal women included in the studies are indicated. In studies where menopausal status was not given, women under the age of 50 were defined as premenopausal and women over the age of 50 as postmenopausal*.

## Hormonal Modulation of Breast Cancer Biomarkers

The abundance of ER fluctuates in normal breast tissue during the menstrual cycle, with ER positivity higher during the follicular phase, compared to the luteal phase (136, 137). Similarly, ER positivity in breast cancers is significantly higher in the follicular phase, when progesterone is absent, compared to the luteal phase (138, 139). This suggests that hormonal fluctuations during the menstrual cycle alter expression of hormone receptors and are highly likely to affect expression of genes associated with hormone signaling.

If menstrual cycle stage affects hormonal receptor signaling and the expression of genes and proteins used in subtype diagnosis, it is expected that there would be discrepancies between diagnostic and surgical breast cancer samples, which would be taken from the woman at different times and, therefore, different stages of the menstrual cycle. However, studies on concordance between biopsy and surgical samples have not specifically investigated premenopausal women. In 2013, Dekker et al. assessed the concordance of ER and HER2 expression between core needle biopsy and surgical resections (140). A concordance of ER was found in 99.1% of patients, and in 96.4% of cases for HER2. The menopausal status of women was not specified in the study; however, the mean age of women enrolled was 63 years old, which suggests that a majority of patients were postmenopausal. In a pooled study of 2507 invasive breast tumors, predominantly from postmenopausal women, concordance of ER was found for 93.4% of patients, and 97.8% for HER2 (140). This high concordance in receptor status led the authors to conclude that ER and HER2 status can be reliably determined from the core needle biopsy. However, these studies focused primarily on postmenopausal women, and the effect of premenopausal factors was not investigated.

In addition to studies investigating concordance in traditional biomarkers, several studies have evaluated the gene expression changes between core biopsies and surgical excisions (141–143). In 2012, Riis et al. compared whole gene-expression profiles of 13 women (1/13 premenopausal; 12/13 postmenopausal) and identified 228 genes differentially expressed, a majority of which are immunoregulatory or stress related (142). Two genes from the PAM50 signature gene list had differential expression between samples; *GRB7* and *ACTR3B*. From the Oncotype DX test, only one gene showed differential expression, *GRB7*. Additionally, Jeselsohn et al. compared gene expression between core biopsies and surgical excisions in postmenopausal women with ER-positive breast cancer (141). The authors identified significant changes in the expression level of 14 genes, a majority of which are immunoregulatory. Two genes involved in the Oncotype DX test and PAM50 intrinsic classification, *MYC* and *CCNB1*, showed differential expression between core biopsies and surgical excisions. A recent study utilized a genome-wide approach to determine gene expression changes between core biopsies and surgical excisions. The authors collected 56 paired core-cuts from postmenopausal breast cancer patients, and classified tumors into one of the five intrinsic subtypes based on the PAM50 gene signature (143). No systematic differences in categorization of the tumors into intrinsic subtypes were identified; however, discordances were identified between the Luminal A versus Luminal B subtype. While these studies have generally found good concordance between diagnostic and surgical samples, concordance in premenopausal women has not been specifically investigated.

A change in biomarker status can have important clinical consequences for adjuvant treatment. Studies evaluating the concordance of hormone receptors and gene expression profiles between core biopsies and surgical excisions suggest that biopsies taken at diagnosis are representative of the whole tumor. However, these studies were performed predominantly again in postmenopausal women, and it is possible that hormonal fluctuations in premenopausal women may alter the expression of these biomarkers. As such, the biopsy taken at diagnosis or during surgery may be influenced by fluctuating concentrations of estrogen and progesterone and, therefore, not represent the true tumor profile. This can lead to an incorrect diagnosis and risk prediction and sub-optimal treatment of premenopausal women.

A recent study compared the expression of estrogen-related genes between pre- and postmenopausal women with ER-positive breast cancer (144). It was suggested that the different hormonal environments of pre- and postmenopausal women may affect the biological characteristics of the breast tumor. The authors found that expression of estrogen-related genes PgR, TFF1, and GATA3, were significantly higher in premenopausal women compared to in postmenopausal women. Consistent with this, studies have also shown that expression of estrogen-regulated genes is significantly associated with the level of estrogen in the blood (145, 146). It is likely that the fluctuating concentration of estrogen during the menstrual cycle affects the expression of these estrogen-related genes. In 2013, Haynes et al. compared the expression of estrogen-related genes between women at different stages of the menstrual cycle (145). They found that the expression of key estrogen-related genes was highest during the follicular phase of the menstrual cycle when estrogen concentration peaks. However, it remains unknown how menstrual cycling can affect the expression of these genes within the same tumor. As Oncotype DX and Prosigna rely heavily on the expression of estrogen-related genes for diagnosing breast cancer, changes in expression of these genes across the menstrual cycle may affect the diagnosis of breast cancer by these tests.

Despite the known role of estrogen and progesterone on the function of the breast and on breast cancer risk, the effect of menstrual cycling on breast tumors remains unknown. In support of the possibility that menstrual cycle critically affects the gene-expression profile of breast cancers, a recent *in vitro* study has suggested that the combination of estrogen and progesterone results in the switching from a Luminal A to Basal-like intrinsic subtype in breast cancer cells, and increases the Oncotype DX Recurrence Score (43) compared to estrogen treatment alone. Tests that utilize gene expression profiling in breast cancer classification were developed and validated from studies predominantly in postmenopausal women, and there is a scarcity of research on how applicable these biomarkers are to premenopausal women, and the extent to which this impacts on treatment response. It is important to understand how hormonal fluctuations affect predictive and prognostic biomarkers, to provide premenopausal women with the optimal treatment for their individual cancer.

## Conclusion

Breast cancer clinics are increasingly adopting gene expression profiling to subtype tumors and identify the best therapies. However, despite their availability to young women, such tests were largely developed and validated in postmenopausal women – patients in whom fluctuations in estrogen and progesterone associated with the menstrual cycle are absent. Yet, these hormones are highly likely to affect breast cancer gene expression in premenopausal women – and the diagnosis and treatment trajectories that stem from its measurement – could fundamentally depend on a patient’s menstrual cycle stage at the time of tissue sampling. Leading diagnostic tests harness intrinsic subtyping of breast cancers, but whether these tests are accurate for premenopausal women remains a startlingly open question. Quite simply, young women may be at risk of receiving inaccurate subtype diagnoses; with ramifications spanning inaccurate prognoses, suboptimal and unnecessary treatments, and reduced survival.

## Author Contributions

All authors contributed intellectually to the development and writing of the manuscript.

## Conflict of Interest Statement

The authors declare that the research was conducted in the absence of any commercial or financial relationships that could be construed as a potential conflict of interest.
